# The Haptoglobin-CD163-Heme Oxygenase-1 Pathway for Hemoglobin Scavenging

**DOI:** 10.1155/2013/523652

**Published:** 2013-05-27

**Authors:** Jens Haugbølle Thomsen, Anders Etzerodt, Pia Svendsen, Søren K. Moestrup

**Affiliations:** Department of Biomedicine, University of Aarhus, Ole Worms Alle 3, Building 1170, 8000 Aarhus C, Denmark

## Abstract

The haptoglobin- (Hp-) CD163-heme oxygenase-1 (HO-1) pathway is an efficient captor-receptor-enzyme system to circumvent the hemoglobin (Hb)/heme-induced toxicity during physiological and pathological hemolyses. In this pathway, Hb tightly binds to Hp leading to CD163-mediated uptake of the complex in macrophages followed by lysosomal Hp-Hb breakdown and HO-1-catalyzed conversion of heme into the metabolites carbon monoxide (CO), biliverdin, and iron. The plasma concentration of Hp is a limiting factor as evident during accelerated hemolysis, where the Hp depletion may cause serious Hb-induced toxicity and put pressure on backup protecting systems such as the hemopexin-CD91-HO pathway. The Hp-CD163-HO-1 pathway proteins are regulated by the acute phase mediator interleukin-6 (IL-6), but other regulatory factors indicate that this upregulation is a counteracting anti-inflammatory response during inflammation. The heme metabolites including bilirubin converted from biliverdin have overall an anti-inflammatory effect and thus reinforce the anti-inflammatory efficacy of the Hp-CD163-HO-1 pathway. Future studies of animal models of inflammation should further define the importance of the pathway in the anti-inflammatory response.

## 1. Introduction

Erythrocytes produced in the bone marrow have a life span of average 120 days [1]. At this time the senescent erythrocytes have undergone changes in the phospholipid composition in the plasma membrane and they are recognized and phagocytosed by macrophages particularly in the spleen red pulp and the bone marrow [2]. Alternatively, the erythrocytes can rupture in the circulation before their expected recognition by the macrophages. This intravascular hemolysis accounts for about 10–20 percent of the total turnover of erythrocytes during normal physiological conditions. Several diseases such as hemoglobinopathies, autoimmune disorders, malaria, and other infections may highly increase intravascular hemolysis thus challenging the inherent Hb scavenging from plasma [3]. Hb can also be released outside the vascular system in case of internal bleedings such as microvascular and macrovascular hemorrhage. The Hb release due to intravascular hemolysis and internal bleeding may have damaging effect on the tissues [4].

**Figure 1:**
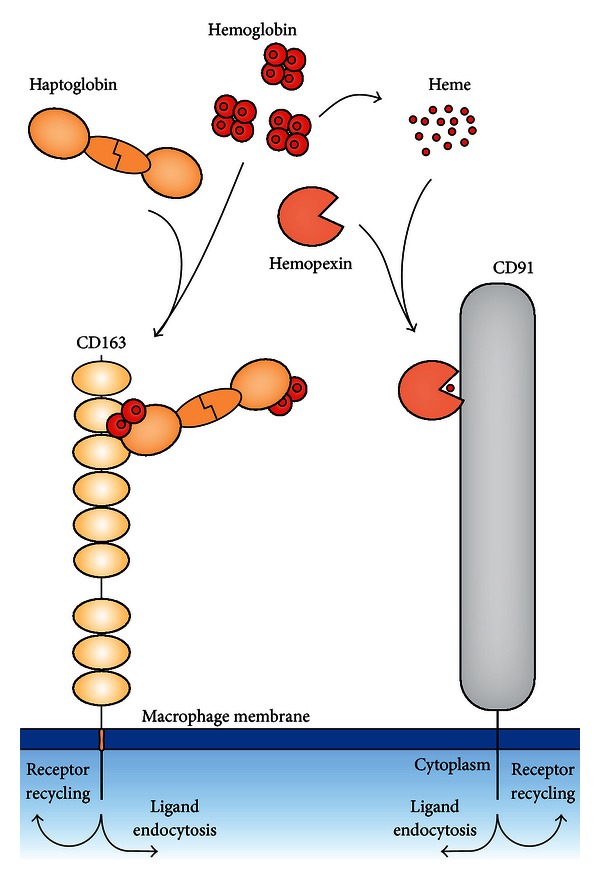
The CD163 pathway for uptake of Hb-Hp complexes and the CD91 pathway for uptake of hemopexin- (Hx-) heme complexes. The endocytosis of the ligand leads to degradation in lysosomes while the receptor recycles from the endosomes back to the plasma membrane.

The Hb-binding Hp represents a first defense line that instantly reduces the Hb toxicity and facilitates its removal by CD163 (Figure 1). This leads to proteolytic degradation of Hb and catabolism of the toxic heme moiety via the HO-1 pathway. In this review, we describe the proteins in this pathway and their suggested role in the anti-inflammatory response.

## 2. Hp—Expression, Structure, and Binding of Hb

Hp is an abundant plasma glycoprotein (0.3–3.0 g/L) secreted primarily by hepatocytes but also by other cell types, such as monocytes/macrophages and neutrophils [4–6]. It is posttranslationally cleaved into an *α*- and a *β*-chain forming a complement control protein (CCP) domain and a serine proteinase domain, respectively [7]. The two domains remain connected through disulfide bridges. Furthermore, the CCP *α*-chain connects to another *α*-chain leading to the Hp *α*2*β*2 formation, which is the basic form seen in all species. Higher polymeric forms are also seen in humans because of the two allelic Hp variants genes Hp1, and Hp2 [8]. They give rise to three possible phenotypes: Hp1-1, Hp2-1 and Hp2-2. The Hp2 gene contains a duplication of a part of the Hp1 gene, which results in a Hp protein with a duplicated *α*-chain. This causes the formation of a range of polymeric forms of the Hp2-1 and Hp2-2 phenotypes [5]. The phenotype is associated with slight differences in average plasma Hp levels (Hp1-1 > Hp2-1 > Hp2-2) [9]. 

Hp has different expression patterns in mammals and responds to various degrees on inflammation. In humans, Hp is moderately upregulated during acute phase conditions, where the acute phase mediators interleukin-1 (IL-1) and IL-6 further stimulate Hp synthesis in hepatocytes [5, 10]. At sites of inflammation, Hp may be upregulated locally by release from stored granules in activated neutrophils [6].

Recent determination of the crystal structure of the porcine Hb-Hp complex revealed a barbell-like structure with oxygenated Hb bound to the serine proteinase domain (*β*-chain) of Hp. In this structure, the CCP domains were connected by the formation of a not previously described CCP fusion domain formed by *β*-strand swapping [7]. The binding interface encompasses several of the amino acids prone to oxidation in the absence of Hp, thus providing a structural basis for the direct protective function of Hp [11]. The “loop 3 region” of the serine proteinase domain previously shown to be involved in the binding of Hp to CD163 protrudes from the complex [7]. 

Hb released into plasma from ruptured erythrocytes dissociates into dimers that instantly bind to Hp by a virtually irreversible interaction [3]. It thereby directly “detoxifies” the oxidative Hb, prevents its filtration in the kidney [5, 12], and promotes the CD163-mediated uptake of Hb in macrophages [13]. 

Hb's toxicity relates in particular to the formation of oxygen radicals and the scavenging of nitric oxide (NO) [11]. The iron coordinated in Hb and heme reacts strongly in the presence of hydrogen peroxide producing hydroxyl radicals and downstream oxidation products. While sequestered in the erythrocytes, cytoplasmic enzymes such as catalase and superoxide dismutase limit the concentration of hydrogen peroxide and oxygen anions and thus the oxidative reactivity of Hb. When bound to Hp, the oxidative intermediate Hb-Fe^IV^ is stabilized and rendered less kinetically active [14]. Additionally, Hp protects Hb from oxidative modifications that would otherwise prevent its clearance or result in release of free heme to the circulation [15, 16]. Binding of NO to Hb in plasma impairs NO signaling, which may affect platelet aggregation and increase vascular contraction [3]. These are serious symptoms in diseases with strong hemolytic crises such as sickle cell anemia. Hp has not yet been shown to protect against NO scavenging directly, but the observed protection provided by Hp against nitric oxide scavenging is probably due to the accelerated clearance of Hb as mediated by Hp [17].

The binding of Hb to Hp1-1 leads to the formation of an approximate 160 kDa complex. Much larger complexes are formed, when Hb binds to the Hp2-1 and Hp2-2 forms. Whatever kind of Hb-Hp complex is formed, the complex formation effectively reduces renal filtration of Hb [5, 12]. In addition, it elicits a high affinity site for CD163 recognition leading to clearance of Hp and Hb [18]. As a consequence, hemolysis leads to consumption of Hp that can be virtually absent, if the release of Hb into plasma overrides the production of the Hp. A low Hp level in plasma is therefore a strong and well-known biomarker for accelerated intravascular hemolysis. Despite circulating Hp in its free none-Hb-bound form does not bind to CD163, the Hb-bound Hp is directly involved in the binding to CD163. Extensive mutagenesis studies of Hp have identified basic residues in Hp loop 3 as important residues involved in the receptor binding [19, 20]. It is yet not known if Hb binding is involved in CD163 binding of the Hp-Hb complex.

## 3. Other Roles of Hp

Besides its established effect in protecting against the toxic effect of Hb, other functions of Hp have been reported. These functions, which are yet less explored than the Hb-related function of Hp, include promoted angiogenesis and an overall anti-inflammatory effect as reviewed elsewhere [5, 12]. Furthermore, speculations of other roles of Hp are nourished by intriguing correlations between various diseases and Hp phenotypes (reviewed by Levy et al. [5]). In diabetic patients, the risk of cardiovascular disease is reported significantly higher for patients with the Hp2-2 genotype [21]. In the same group of patients, vitamin E supplementation has been shown to be protective against these cardiovascular complications [22]. Studies of cases of subarachnoid hemorrhage also indicate an increased risk of cerebral vasospasms in Hp2-2 individuals compared to Hp1-1. On the other hand, Hp2-2 has been proposed to have a protective function against malaria [23, 24]. A recent study demonstrates a link between hemolysis-induced activation of the HO-1 and neutrophil dysfunction which may be affected by the Hp concentration and phenotype [25]. However, it should be noted that other malaria studies have not confirmed significant association of Hp genotype on disease outcome [26–28]. Further epidemiological analyses and biochemical studies are warranted to document and mechanistically understand associations between Hp phenotype and disease. 

## 4. CD163—Structure, Expression, and Receptor Function for Hp-Hb

CD163 is a 130 kDa transmembrane glycoprotein expressed exclusively in cells of the reticuloendothelial system. It is a member of the “scavenger receptor cysteine rich” (SRCR) superfamily class B. This receptor family is characterized by containing one or more SRCR domains that are conserved domains consisting of 100–110 amino acids and 6 to 8 cysteine residues connected by disulfide bridges [29]. Crystallization of the repeat in other proteins has revealed a compact fold of 5-6 *β*-sheets cradling an *α*-helix [30–32]. Class A and class B SRCR domains are structurally similar with only a few slight differences. Class B domains are translated from a single exon, and class A domains are from two exons and they contain one more disulfide bridge than class B [33]. The extracellular segment of CD163 contains nine SRCR domains only separated by a 34 proline/serine/threonine-rich linker region between domain 6 and 7 [34]. 

Four different isoforms have been demonstrated, resulting from alternative splicing of the RNA encoding the cytoplasmic tail [35]. The shortest and most abundant variant consists of 49 amino acid residues, while the longest consists of 84 and 89, respectively. The first 42 amino acids after the transmembrane segment are identical amongst the isoforms and contain phosphorylation motives for casein kinase and protein kinase C [36]. Other possible phosphorylation motives are present on the longer isoforms [33]. Confocal microscopy has revealed that the shortest tail variant is primarily present in the cell membrane while the longer variants are located in the endosomal/Golgi cellular compartment [37]. 

CD163 is expressed exclusively on cells of the monocyte-macrophage cell lineage. A high expression is seen in most mature tissue macrophages such as Kupffer cells in the liver, red pulp macrophages in the spleen, resident bone marrow macrophages, and alveolar macrophages in the lungs [34]. Cell types derived from monocytes showing low or no CD163 expression include dendritic cells, Langerhans cells, and white pulp macrophages in the spleen [38, 39].

Several endogenous and exogenous molecules have been shown to regulate the expression of CD163 in *in vitro* experiments. Glucocorticoids, IL-6, and interleukin-10 (IL-10) strongly upregulate its expression, whereas interferon-*γ* (IFN*γ*), tumor necrosis factor-*α* (TNF-*α*), interleukin-4 (IL-4), granulocyte/macrophage colony stimulating factor (GM-CSF), lipopolysaccharide (LPS), and CXC-chemokine ligand 4 (CXCL4) downregulate CD163 expression [13, 34, 35]. The upregulation of CD163 by glucocorticoids has also been demonstrated in human volunteers following injection with the glucocorticoid prednylidene [40]. Whereas IL-6 has both pro- and anti-inflammatory effects [41], the overall pattern is that CD163 expression is induced by anti-inflammatory mediators and reduced by proinflammatory molecules.

Experimental studies have shown that CD163 is expressed on macrophages matching the phenotype defined by *in vitro* differentiation in response to IL-4 and interleukin-13 (IL-13) (M2/alternatively activated macrophages) despite the fact that IL-4 alone decreases CD163 expression in monocyte/macrophages [34, 42]. CD163 positive macrophages of a similar phenotype are abundant in the resolution phase of the inflammatory process [43]. These findings have been used to hypothesize that CD163 is a marker of an anti-inflammatory and tissue homeostatic macrophage subclass [13]. CD163 is now widely used as a marker for the macrophage class. Finally, a novel macrophage subtype designated Mhem is defined by a high CD163 and a low mannose receptor expression [44]. These macrophages have been described in atherosclerotic lesions and they were suggested to exhibit an antiatherogenic phenotype when examined *in vivo* [45]. 

A soluble form of CD163 (sCD163) is present in plasma and it is upregulated in a number of diseases involving macrophages as recently reviewed by Moller [46]. It is generated by ectodomain shedding of the extracellular part of the receptor. Both TNF-*α* cleaving enzyme (TACE)/ADAM17 and neutrophil elastase have been reported as enzymes responsible for the cleavage [47]. However, the concomitant increase in sCD163 and TNF-*α* in humans exposed to LPS does suggest an important role of TACE/ADAM17, which is activated by LPS in macrophages [47]. The biological function of sCD163 is not yet clear, although several possible functions have been proposed—including opsonization of Staph. Aureus [48], inhibition of T-cell proliferation [49] and inhibition of tumor necrosis factor-like weak inducer of apoptosis (TWEAK) [50]. 

The third SRCR domain of CD163 is involved in the Ca^2+^-dependent binding of the Hp-Hb complex [18, 51]. The subsequent endocytosis of the ligand bound receptor is dependent on the endocytic motifs in the cytoplasmic tail [37, 52]. The various CD163 isoforms differ in endocytic efficacy with the shortest variant demonstrating the fastest uptake [37]. 

In addition to its uptake of the Hb-Hp complex CD163 can facilitate the uptake of free Hb. This allows CD163 to act as its own fail-safe system in pathological situations where Hp is depleted due to excessive intravascular hemolysis [53]. To what degree this function is implicated in human disease is unknown. Studies in the mouse Hp-Hb system have disclosed subtle differences. In this species, Hb binds with higher affinity to CD163 and the binding of Hp to Hb does not further increase affinity for CD163 [54]. The CD163-mediated uptake of Hb (in complex with Hp or not) induces the secretion of IL-6 and IL-10, as well as it upregulates several genes responsible for the degradation of Hb-including HO-1 [36, 55]. 

## 5. Other Potential Functions of CD163

Several functions besides the scavenging of Hb have been proposed for CD163. In rats, CD163 expressed on resident bone marrow macrophages has been shown to bind erythroblasts and promote growth and/or survival in erythropoiesis [56]. A recent study indicates a role of CD163 as a pathogen-associated molecular pattern (PAMP) receptor [57]. CD163 demonstrated binding to both gram-positive and negative bacteria and the bacteria induced TNF-*α* secretion from human monocytes [57]. TWEAK has been shown to be bound and internalized by CD163, indicating CD163 as a possible regulator of this cytokine [58]—in addition to the regulation of TWEAK by sCD163 as mentioned earlier. A high sCD163 and low TWEAK concentration has been shown to correlate with intima-media thickness, cardiovascular mortality in peripheral arterial disease, and a type 1 diabetes diagnosis [50, 59, 60]. Finally, porcine CD163 has been implicated in the entry mechanisms of African swine fever virus (ASFV) and the porcine reproductive and respiratory syndrome virus (PRRSV) infecting myeloid cells [61, 62].

## 6. Physiological Back-Up Systems for the Heme-Protective Function of the Hp-Hb Pathway

Excessive hemolysis as seen during malaria, sickle cell anemia, autoimmune hemolysis, and many other conditions with pathological intravascular hemolysis may lead to depletion of Hp in plasma [63]. In such cases, Hb accumulates in plasma with toxic consequences. Hb may then be taken up directly by CD163 by a yet unknown pathway, be filtered in the kidney or be degraded in plasma. The absence of Hp binding to Hb leads to release of heme that then binds to heme-binding proteins such as albumin, *α*1-microglobin, and hemopexin. Hemopexin binds heme with the highest affinity leading to uptake via LDL-receptor related protein 1 (LRP) [64]/CD91 (Figure 1), an abundant receptor in macrophages, hepatocytes and other cells [65]. Studies of hemopexin-deficient mice with and without a Hp gene knockout background have evidenced that hemopexin constitutes a backup system for the heme-protective role of Hp [66]. 

## 7. HO-1

**Figure 2:**
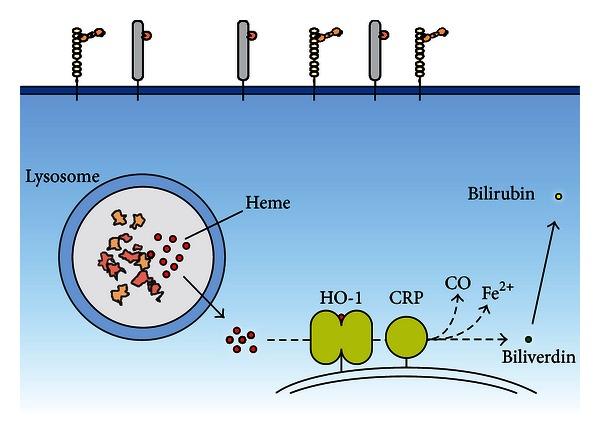
The intracellular pathway for heme-degradation subsequent to CD163 and CD91 mediated endocytosis in macrophages. Free heme is degraded to biliverdin, CO, and ferrous iron by the endoplasmic reticulum enzyme HO-1 facing the cytosol. Electrons are delivered by NADPH p450 cytochrome reductase. Biliverdin is reduced to bilirubin by biliverdin reductase and transported to the liver bound to albumin.

Hb internalized through interaction with CD163 is transferred to early endosomes and subsequently degraded to heme, bioactive peptides, and amino acids [52, 67]. HO is responsible for the further enzymatic heme catabolism resulting in the degradation products carbonmonoxide (CO), ferrous iron (Fe^2+^) and biliverdin. Biliverdin is reduced to bilirubin by the biliverdin reductase (Figure 2). 

Three isoforms of HO differing in tissue distribution, regulation and proposed function have been identified. HO-1 (33 kDa) is expressed in many cell types including macrophages. It is highly inducible in response to a wide range of factors [68]. HO-2 (36 kDa) is constitutively expressed with the highest expression in testis and brain [69]. Finally, an HO-3 isoform was identified in rats but later studies suggest it may be a pseudogene with no apparent function [70, 71].

HO-1 is a monomeric enzyme anchored to the outer membrane of a microsomal membrane by a hydrophobic C-terminal domain [72]. More recently HO-1 has also been identified in caveolae demonstrating direct interaction with caveolin-1 [73]. Whether the heme oxidation takes place in the cytosol or in endosomal vesicles is not fully outlined [74]. Proteolytic cleavage of the active site of HO-1 from the membrane-anchored C-tail occurs following hypoxia or heme loading leading to translocalization of the truncated enzyme to the nucleus. Here it promotes transcription of antioxidative related genes including activating protein-1 (AP-1) [75]. The binding site for heme is located between two concave *α*-helixes termed the proximal and distal helix, respectively [76].

The enzymatic process leading to the degradation of heme comprises three major steps. In the first step heme is oxidized to hydroxyheme, and in the second step verdoheme is formed and CO is released. The third step results in biliverdin and Fe^2+^ [77]. The last step is rate limiting but is also the least characterized [78]. During the process three molecules of oxygen and seven electrons are consumed [76]. The electron donor is NADPH cytochrome p450 reductase, which is anchored alongside HO-1 on the endoplasmic reticulum on the side facing the cytosol [79].

The molecular mechanisms for the regulation of HO-1 have been extensively investigated and the complexities of the pre-translational regulation are now steadily being unraveled although hampered by major differences between the examined species and between cell types [80, 81]. The expression of HO-1 is inducible by a long list of endogenous and exogenous molecules [80]. In the context of this review, it should be noted that heme itself aside from functioning as cofactor and substrate of HO-1 also seems as the most potent inducer of HO-1 expression. Other inducers include, but are far from being limited to, heat, ultraviolet radiation, LPS, hydrogen peroxide, several dietary phytochemical, IL-1*α*, TNF*α*, and NO [82–88]. Interestingly, IL-10, which has a central role in the CD163 regulation, also stimulates synthesis of HO-1 [89]. Some common mechanisms have been proposed based upon shared cellular effects of some the inducers: a transient increase in intracellular heme, increased production of reactive oxygen species (ROS) generation, and glutathione depletion [80]. 

Many studies have demonstrated a role for protein phosphorylation dependent signaling pathways in the observed HO-1 upregulation. A growing body of evidence points to a central role for the mitogen associated protein kinases (MAPK) family of kinases in this. MAPK proteins belong to the serine/threonine kinase superfamily and is involved in mediating signals for cell growth, differentiation, and apoptosis and commonly activated in response to stressors [90]. PI3 K/Akt, protein kinase A, protein kinase C, and tyrosine kinase have also been implicated as possible mediators of HO-1 induction [80]. 

The existence of multiple HO-1 inducers corresponds with the abundance of response elements and cis-acting elements in the promoter region of Hmox-1 (the human HO-1 gene). The promoter region spans at least 11 kb from the 5′ start of Hmox-1 and contains several consensus binding motives for binding of transcription factors such as ARE (antioxidant response element or stress-related response element) which is found in the promoters of proteins associated with anti-oxidative functions (also known as phase II enzymes) [91–93]. Its ligands include transcriptions factors of the basic leucine zipper-superfamily of which several have been shown to induce HO-1 transcription. NF-E2 related factor 2 (Nrf-2) belongs to this family as well and a growing body of evidence shows that it is essential for ARE-binding and HO-1 induction [80, 94].

Until recently, no direct molecular link between increased oxidative stress and transcriptional activity was known. The identification of the interactions between Nrf-2 and Kelch-like ECH associated protein-1 (Keap-1) has provided such a link. Under low-stress conditions Keap-1 binds Nrf-2 in the cytoplasm and directs it to ubiquitin-dependent proteasomal degradation [94, 95]. Oxidation of specific cysteine residues in Keap-1 or the phosphorylation of Nrf-2 inhibits its degradation and result in nuclear translocation, heterodimerization, and transcriptional activity of Nrf-2 [94]. This model explains both the observed link between MAPK activation and Nrf-2 transcriptional activity [96, 97] and the link between oxidative stress/ROS and HO-1 expression. Using knock-out technology and genetic transduction a protective role of HO-1 in a variety of disease models including atherosclerosis, hypoxia, hyperoxia-induced lung damage, liver failure, liver allograft, hypertension and reperfusion injury has been shown [98–105].

## 8. The Physiological Effects of Heme Metabolites

CO is most widely known as a toxic inhaled gas inhibiting oxygen binding and release from Hb thus causing asphyxiation. However, as mentioned earlier, it is also endogenously produced during heme oxidation by HO-1. At these relative low levels an increasing body of evidence indicates that CO serves several beneficial physiological functions. Most of these cellular effects are believed to be mediated by CO binding to heme in heme-proteins [106]. Of notice, CO binds and activates soluble guanylate cyclase (sGC) to produce cyclic guanosine monophosphate similarly to nitric oxide though with less efficacy [107]. This may mimic nitric oxide's well-established cytoprotective effects and this is believed to be a major contributor to the observed cytoprotection mediated by HO-1/CO [80, 108]. 

CO has also been shown to cause vasodilatation via sGC independent activation of potassium channels in vascular smooth muscle cells [109]. Furthermore, CO is believed to modulate p38 MAPK in an sGC independent way by inhibiting the expression of classical pro-inflammatory cytokines such as TNF-*α*, IL-1*β*, and macrophage inflammatory protein-1*β* while promoting the expression of the anti-inflammatory cytokine IL-10 [110]. These anti-inflammatory effects have been demonstrated *in vivo* where administered CO in nonlethal concentrations was able to reduce the inflammation induced by mechanical ventilation [111, 112]. Cytochrome c oxidase (COX) is a heme-protein in the inner mitochondrial membrane which transfers protons and electrons to O_2_, creating water and providing energy for transport of two protons across the membrane. CO binds and inhibits this protein thus inhibiting O_2_ consumption and stimulating ROS from the accumulating electron carriers in the mitochondria. This process is termed mitochondrial redox signaling, and it is believed to stimulate mitochondrial biogenesis and angiogenesis [106]. 

The CO cleaved from hydroxyheme is primarily removed from the body via respiration. CO diffuses readily cross-cell membranes and binds Hb with approximately 200-fold higher affinity than O_2_. Provided adequate circulation and respiration, it is then transported to the alveoli and diffuses to the alveolar gas. Additionally, CO is slowly oxidized by COX to CO_2_ [106]. The exhalation of CO can be used as a measure of heme catabolism in the body [113].

Iron (Fe^2+^) is released from heme during the last enzymatic step of its conversion to biliverdin. A P-type ATPase iron transporter is colocalized with HO-1 in the microsomal membrane [114]. The importance of this iron release is suggested because of the anemia and iron accumulation in the liver and kidney in HO-1 knock-out mice and in the first reported case of human HO-1 deficiency [80, 115, 116]. Most likely, the iron released from heme enters a labile pool of intracellular iron, available for cellular processes involving iron or cellular export via the hepcidin-regulated ferroportin protein in the membrane.

Intracellular iron is oxidized and bound to the ubiquitous apoprotein ferritin. An increase in the intracellular iron deposit affects the posttranscriptional expression of several proteins by interaction with iron regulatory proteins and mRNA iron response elements [117]. Via this mechanism ferritin is upregulated by increased HO-1 activity [118]. Ferritin has been shown to have antiapoptotic effects and provide cytoprotection against oxidative damage [119, 120]. Iron is exported from the cell by ferroportin and transported bound to the plasma protein transferrin. Transferrin-bound iron is taken up by cells expressing the transferrin receptor and recycled.

The main product of heme degradation, biliverdin, is a greenish water-soluble pigment. It is reduced to bilirubin by biliverdin reductase. Bilirubin is a hydrophobic, yellowish pigment and is transported in the plasma bound to albumin. In the liver, bilirubin is conjugated and excreted in the bile. For decades bilirubin has been considered a toxic byproduct of heme degradation. Recent studies have, however, also demonstrated potential beneficial functions of bilirubin and biliverdin in the circulation and extravascular tissues [121]. Epidemiological studies have revealed that moderately increased plasma levels of bilirubin decrease the risk of developing cardiovascular diseases [122]. *In vitro* studies have demonstrated bilirubin and biliverdin as functional antioxidants [123]. Biliverdin reductase has also been shown to be at least partly responsible for HO-1-mediated anti-oxidative protection [124]. Additionally, biliverdin reductase has been shown to promote an anti-inflammatory response in macrophages through transcriptional regulation [125].

## 9. Perspectives

The Hp-CD163-HO-1 pathway for degradation of hemoglobin is as an important and apparently to some extent a coordinately regulated pathway that by direct hemoglobin binding and subsequent clearance from plasma prevents toxic and proinflammatory effects of heme and hemoglobin. In addition, the proteins in the pathway and the metabolic heme products reinforce an anti-inflammatory response.  Table 1 summarizes major anti-inflammatory effects of this pathway. Future studies of various inflammatory conditions *in vitro* and *in vivo* models should further delineate the molecular mechanism and elucidate if the proteins of the pathway have anti-inflammatory effects independent of heme. Finally, this pathway seems as a potential target for stimulation of the inflammatory response by small molecule drugs.

## Figures and Tables

**Table 1:** Major reported cytoprotective and antiinflammatory effects of the Hp-CD163-HO-1 pathway.

Intravascular HpHb complex formation:
Protects against oxidative “hot spot” in Hb
Protects against heme release from Hb
Facilitates CD163-mediated clearance
Prevents renal filtration of Hb and uptake in proximal tubules
Prevents NO scavenging
Cellular response on CD163-mediated Hb endocytosis:
Cellular differentiation
HO-1 upregulation
Nrf-2 activation
IL-10 synthesis
Other effects of heme metabolites generated by HO-1 activity:
Antagonism of proinflammatory cytokines
ROS scavenging
Angiogenesis
Inhibition of platelet aggregation
Vasodilatation
